# BubR1 as a prognostic marker for recurrence-free survival rates in epithelial ovarian cancers

**DOI:** 10.1038/sj.bjc.6605161

**Published:** 2009-07-14

**Authors:** Y-K Lee, E Choi, M A Kim, P-G Park, N-H Park, H Lee

**Affiliations:** 1 Department of Biological Sciences and Research Center for Functional Cellulomics, College of Natural Science, Seoul National University, San 56-1, Shinlim-dong, Gwanak-ku, Seoul 151-742, Republic of Korea; 2 Department of Obstetrics and Gynecology, College of Medicine, Seoul National University, Seoul, Republic of Korea; 3 Department of Pathology, College of Medicine, Seoul National University, Seoul, Republic of Korea

**Keywords:** BubR1, Aurora A, ovarian neoplasm, prognosis, recurrence-free survival

## Abstract

**Background:**

Epithelial ovarian cancer is one of the most lethal malignancies, and has a high recurrence rate. Thus, prognostic markers for recurrence are crucial for the care of ovarian cancer. As ovarian cancers frequently exhibit chromosome instability, we aimed at assessing the prognostic significance of two key mitotic kinases, BubR1 and Aurora A.

**Methods:**

We analysed paraffin-embedded tissue sections from 160 ovarian cancer patients whose clinical outcomes had been tracked after first-line treatment.

**Results:**

The median recurrence-free survival in patients with a positive and negative expression of BubR1 was 27 and 83 months, respectively (*P*<0.001). A positive BubR1 expression was also associated with advanced stage, serous histology and high grade. In contrast, Aurora A immunostaining did not correlate with any of the clinical parameters analysed.

**Conclusion:**

BubR1, but not Aurora A, is a prognostic marker for recurrence-free survival rates in epithelial ovarian cancers.

Epithelial ovarian cancer is a highly lethal malignancy (Jemal *et al*, 2007). Although cytoreductive surgery, followed by adjuvant chemotherapy, enhances clinical responses in the majority of ovarian cancer patients, many of them experience a recurrence of disease and eventually die from resistance to chemotherapy (Kristensen and Trope, 1997). Previous studies have reported clinicopathological prognostic parameters for ovarian cancer including stage, grade, remnant of disease, and presence of ascites (NIH Consensus Conference, 1995). However, these factors have limitations in that they fail to predict the chances of survival for individual patients. Biological behaviour of the tumour, response to chemotherapy, and survival of patients vary even among clinically similar cases (Friedlander, 1998). Thus, the identification of individual prognostic factors, such as molecular markers, is required for predicting disease outcome and choice of treatment.

Chromosome instability (CIN), characterised by aneuploidy and spontaneous chromosome aberrations, is a hallmark of aggressive cancers (Sen, 2000). It is noteworthy that aneuploidy is an important feature in ovarian cancers (But and Gorisek, 2000; Shridhar *et al*, 2001; Gorringe *et al*, 2005). Therefore, understanding the basic mechanisms that lead to aneuploidy in ovarian cancer is crucial in determining the appropriate course of treatment.

Aneuploidy can result from inaccurate chromosome segregation, dysregulated centrosome cycles, or improper cytokinesis (Chi and Jeang, 2007). Therefore, the spindle assembly checkpoint (SAC), which ensures that all chromosomes are attached to bipolar spindles and that sister chromatid separation occurs accurately, has a crucial function in genetic integrity. Impaired SAC function has been suggested to be one of the causes of aneuploidy in human cancers (Cahill *et al*, 1998; Amon, 1999). Among the SAC components, BubR1 mitotic kinase, known as BUB1B by the Human Genome Organization, attracts a lot of attention because it is central in the inhibition of Anaphase promoting complex/cyclosome (APC/C) (Tang *et al*, 2001; Yu, 2002; Chan and Yen, 2003; Choi *et al*, 2009). Furthermore, BubR1 monitors kinetochore-microtubule attachments and has a pivotal function in checkpoint signaling (Abrieu *et al*, 2000; Lampson and Kapoor, 2005; Elowe *et al*, 2007).

An accurate centrosome number and organisation is critical in the equal segregation of chromosomes to daughter cells during mitosis. Indeed, centrosome aberrations have been observed in many cancers (Nigg, 2002, 2006). Aurora A kinase is particularly important for centrosome regulation because it is required for the maturation of centrosomes during mitosis (Hannak *et al*, 2001; Berdnik and Knoblich, 2002). Notably, the overexpression or amplification of Aurora A kinase has been found to occur in many human cancers (Zhou *et al*, 1998; Sen *et al*, 2002, 2008; Tong *et al*, 2004), and thus has been suggested to provoke aneuploidy and tumourigenesis (Sen *et al*, 2002, 2008; Tong *et al*, 2004). Although overexpression of Aurora A is clearly responsible for aneuploidy, whether it induces carcinogenesis is not clear (Anand *et al*, 2003; Zhang *et al*, 2004; Giet *et al*, 2005).

In this study, we investigated whether BubR1 or Aurora A levels can be used as prognostic markers for ovarian cancers.

## Materials and methods

### Cell culture of ovarian cancer cell lines and primary ovarian cancers

Human ovarian cancer cell lines, OVCAR3, SK-OV-3, and SNU119, were obtained from the Korean Cell Line Bank and grown in monolayer cultures in RPMI 1640 supplemented with fetal bovine serum (10% v/v).

Primary ovarian cancer samples were collected from the Seoul National University Hospital from patients undergoing cytoreductive surgery. Tumour tissue was dissected into a 100 mm Petri dish containing serum-free RPMI medium supplemented with trypsin. After 30 min at 37°C, the cells were resuspended and maintained in RPMI 1640 supplemented with fetal bovine serum (10% v/v).

### Patients and tissue samples

Tissue specimens for immunohistochemistry were obtained from 160 epithelial ovarian carcinomas by primary cytoreductive surgery between 1998 and 2005. All samples were taken from primary ovarian lesions in each patient. The disease stage of each sample was determined according to the International Federation of Gynecology and Obstetrics (FIGO) criteria. Of the patients, 82 experienced a relapse and 37 died of ovarian cancer. Ten normal ovarian tissue samples were obtained as controls. Formalin-fixed, paraffin-embedded ovarian carcinoma tissues were used for histological evaluation. All haematoxylin and eosin (H & E)-stained sections were reviewed by gynaecological pathologists. The study was approved by the Institutional Review Board.

### Western blot analysis

Protein concentrations were measured with the Bradford protein assay before analysis. Total cell lysate (30 *μ*g of protein) was separated by SDS–polyacrylamide gel electrophoresis and subjected to western blot analysis. Anti-BubR1 antibody (BD, San Jose, CA, USA) was used at a dilution of 1 : 1000 and anti-Aurora A antibody (Transgenic, Japan) at a dilution of 1 : 300.

### Immunofluorescence analysis

Cells grown on cover slips were fixed with 4% paraformaldehyde, then permeabilised with 0.5% Triton X-100 phosphate-buffered saline (0.5% PBS-T). They were then incubated in blocking solution (20% goat serum in 0.1% PBS–T) and probed with anti-BubR1 antibody or anti-Aurora A antibodies. The cells were mounted with Vectashield containing 4′,6-diamino-2-phenylindole (DAPI) (Vector Laboratories, Burlingame, CA, USA). Images were acquired on the DeltaVision microscope (Applied Precision, Seattle, WA, USA) as a series of 0.4-*μ*m-thick sections and merged.

### Cytogenic analysis

Spreads of metaphases were described previously with slight modifications (Patel *et al*, 1998; Lee *et al*, 1999).

### Immunohistochemical analysis

Paraffin blocks from ovarian carcinoma were cut at 4 *μ*m adjacent to H & E sections. Samples were deparaffinised in xylene, rehydrated with graded ethanol, and washed in distilled water. The sections were then placed in 10 mM citrate buffer (pH 6.0) and boiled in a microwave for epitope retrieval. Endogenous peroxidase activity was quenched by incubating tissue sections in 3% H_2_O_2_ for 10 min. After the blocking procedure with 20% goat serum and 1% bovine serum albumin (BSA) in PBS at room temperature for 1 h, sections were incubated with a primary antibody against BubR1 (mouse monoclonal, BD) at a dilution of 1 : 300 or Aurora A (rabbit polyclonal, Transgenic) at a dilution of 1 : 250 in a humidifying chamber at 4°C overnight. They were then washed in PBS for 5 min at room temperature, subsequently stained by the labelled streptavidin biotin (LSAB) method using a Dako LSAB kit (Dako, Glostrup, Denmark), and visualised using 3,3′-diaminobenzidine. The sections were then counterstained with haematoxylin.

To check the nuclear staining of Aurora A, antigen retrieval in 20 mM Tris–EDTA buffer (pH 9.0) was also performed in 80 samples using antibodies from Novocastra (NCL-L-AK2, New Castle, UK) at a dilution of 1 : 50 and following the methods of Burum-Auensen *et al* (Burum-Auensen *et al*, 2007a). The results were similar to the antigen retrieval procedure using citrate (pH 6.0) and Tris–EDTA (pH 9.0) buffer described above (Supplementary Figure). Slides were subjected to the same staining procedure without the addition of primary antibodies for negative controls. Microscopic fields from each stained section were randomly sampled.

In most cases, BubR1 and Aurora A were stained in the cytoplasm in interphase. However, a few specimens revealed nuclear staining of Aurora A, especially when Tris–EDTA buffer (pH 9.0) was used. The percentage of positive staining distribution was recorded and scored as follows: a score of 0 for staining <5%, 1 for 6–25% staining, 2 for 26–50% staining, 3 for 51–75% staining, and 4 for staining >75%. The staining intensity was scored as follows: a score of 0 for absent tumour cell staining, 1 for weak staining (equivocal to normal epithelium), 2 for moderate staining, and 3 for strong staining. For BubR1 and Aurora A, the results for intensity and distribution were summed and total score was assigned as follows: sum of 0–1, score 0; sum of 2–3, score 1; sum of 4–5, score 2; sum of 6–7, score 3.

### Statistical analysis

The relationships between categorical variables were assessed using the *χ*^2^ test. Recurrence-free survival (RFS) was estimated using the Kaplan–Meier method, and the differences in survival were compared using the log-rank test. Multivariate analysis was carried out using the Cox regression method. A *P*-value of <0.05 was considered to be statistically significant. Data were analysed using SPSS software, version 12.0 (SPSS Inc., Chicago, IL, USA).

## Results

### Aberrant BubR1 and Aurora A levels and subcellular localisations in ovarian cancer cells

**Figure 1:**
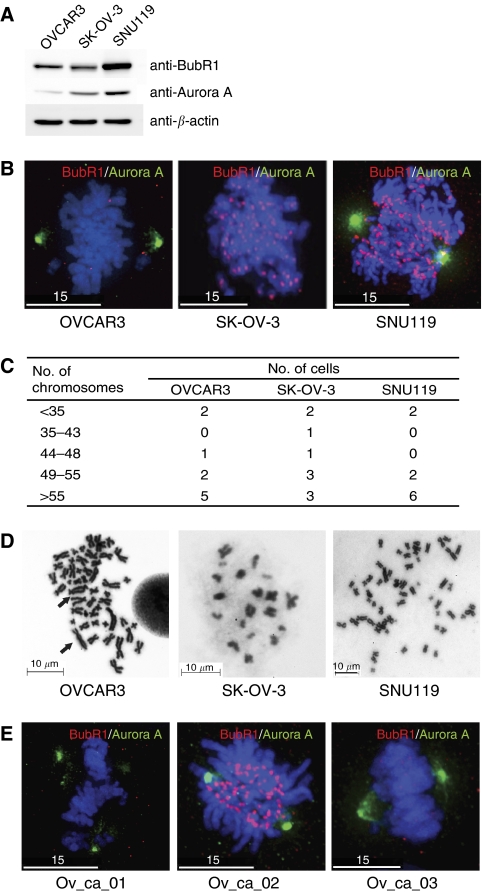
Assessing BubR1 and Aurora A levels and localisation in ovarian cancer cells. (**A**) Western blot analysis of ovarian cancer cell lines, OVCAR3, SK-OV-3, and SNU119, using anti-BubR1 and Aurora A antibodies. (**B**) Immunofluorescence analysis of ovarian cancer cell lines (OVCAR3, SK-OV-3, and SNU119). Cells grown on cover slips were fixed and immunostained with anti-BubR1 (red) and anti-Aurora A (green) antibodies. DAPI (blue) staining reveals chromosomes. (**C**) Cytogenic analysis of ovarian cancer cell lines. Ten different metaphase chromosome spreads were analysed for their chromosome number. All three ovarian cancer cell lines are aneuploid. Diploid cells should have 46 chromosomes. (**D**) Representative metaphase chromosome spreads showing chromosome number aberrations in the three cell lines analysed. In OVCAR3, chromosome fusions, another important measure of CIN, were observed in OVCAR3 and are marked as arrows. Scale bars are marked. **(E)** Immunofluorescence analysis of three primary ovarian cancer cells (Ov_ca_01, Ov_ca_02, and Ov_ca_03). Images were taken and processed on a DeltaVision RT (Applied Precision) with × 1000 magnification. White scale bars 15 *μ*m.

We used western analysis to determine whether the two proteins are expressed in OVCAR3, SK-OV-3, and SNU119 ovarian cancer cells. The results show that all three cancer cell lines, which were randomly picked, express BubR1 and Aurora A to varying degrees (Figure 1A). As accurate mitosis is guaranteed by the proper localisation of BubR1 at kinetochores and of Aurora A at centrosomes, we examined the levels and localisation of BubR1 and Aurora A by immunofluorescence microscopy. As revealed in Figure 1B, all three cell lines analysed displayed defective Aurora A or BubR1 immunostaining at appropriate locations. Furthermore, the patterns of BubR1 and Aurora A staining were heterogeneous from cell to cell in the same cell line (data not shown).

Chromosome instability is accompanied by a heterogeneity of chromosome numbers in individual cells. Therefore, cytogenetic analysis of a statistically significant number of cells is the most direct measure of assessing aneuploidy. To confirm that these three cell lines exhibit CIN, we analysed metaphase chromosome spreads of 10 different cells from each line. The number of chromosomes in all cancer cell lines (OVCAR3, SK-OV-3, and SNU119) was highly variable in 10 different metaphase chromosome spreads (Figure 1C). This result is consistent with information available from the American Culture Type Collection. A representative chromosome spread revealed that one of the cell lines analysed, OVCAR3, exhibited aberrant chromosome fusions, as well as aneuploidy (Figure 1D).

As immunofluorescence assay (IFA) in ovarian cancer cell lines depicted a varying degree of BubR1 or Aurora A levels and subcellular localisation in mitosis (Figure 1B), we next asked whether primary tumour cells exhibit similar results. The result showed that the primary tumour cells also displayed mis-localisations and heterogeneity of BubR1 or Aurora A levels in IFA (Figure 1E).

### Correlation of BubR1 and Aurora A levels and patient survival

Immunohistochemistry staining was conducted on 170 paraffin-embedded samples: 160 ovarian cancer and 10 normal ovary tissues. The age of cancer patients ranged from 18 to 79 years (median, 51.5 years). Table 1 describes the clinicopathological characteristics of 160 cancer patients. Except for 10 patients, all the cancer patients were at stage Ic or higher and hence underwent primary debulking surgery, followed by taxane and platinum chemotherapy. Serous cystadenocarcinoma (57.5%) was the most common histology. Cytoreductive surgery was optimal (⩽1 cm residual disease) in 51.9%. More than two-thirds of patients had a complete response to first-line treatment, yet half of them relapsed (Table 1).

**Figure 2:**
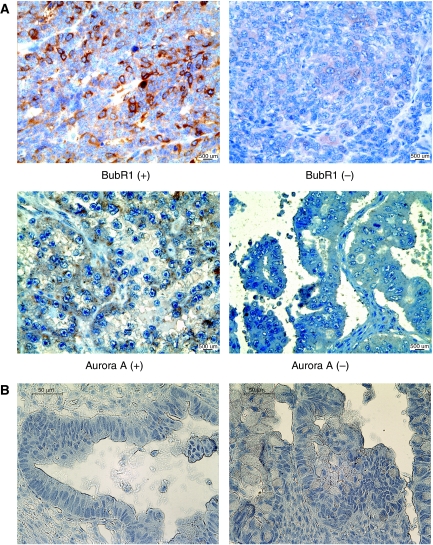
Immunohistochemical staining of BubR1 and Aurora A (**A**) Representative positive and negative immunohistochemistry images of BubR1 and Aurora A in paraffin-embedded human cancer samples (magnification × 400). (**B**) Negative controls for antibody staining. Antigens were retrieved with Tris–EDTA buffer at pH 9.0. IHC was performed without incubation with primary antibodies. Two examples are shown (magnification × 400). Images were taken on an Olympus BX-51 microscope.

**Figure 3:**
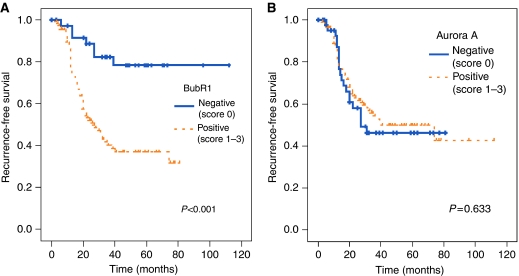
Recurrence-free survival (RFS) using the Kaplan–Meier method in relation to BubR1and Aurora A levels. (**A**) BubR1 positive immunostaining in the tumour had poor RFS compared with negative staining (*P*<0.001). (**B**) Aurora A scores did not show significant correlation with RFS.

Of the 160 ovarian cancer samples, a quarter of them (24.2%) were scored 0 for BubR1 immunostaining and about 30% scored 0 for Aurora A, whereas normal specimens scored 0 in all cases (Table 2, Figure 2). The relationship between levels of BubR1 and the clinicopathological parameters of 160 tumours is shown in Table 3. Positive BubR1 expression was associated with advanced stage (*P*=0.001), serous histology (*P*=0.008), high grade (*P*=0.001), and residual tumour (*P*=0.021). In contrast, no significant correlation was observed between the expression of BubR1 and Aurora A. In univariate survival analysis, BubR1 immunoreactivity was a significant prognostic factor for RFS (*P*<0.001), whereas the Aurora A expression was not (*P*=0.633). Patients with immunoreactive BubR1 (score 1, 2, 3) had a median RFS of 27 months (range, 3–48 months), whereas those with a negative expression of BubR1 (score 0) had a median RFS of 83 months (range, 2–115 months). Figure 3 shows survival curves stratified by BubR1 and Aurora A expression. Multivariate survival analysis (Table 4) indicated that the BubR1 level was an independent prognostic factor for predicting recurrence of ovarian cancer patients.

## Discussion

Strategies in the treatment of ovarian cancers are mainly determined by the degree of tumour differentiation, the FIGO stage of disease, and the volume of residual disease after surgery. However, owing to a significant degree of tumour heterogeneity, even in the same prognostic subgroup, the development of new prognostic markers that can aid in selecting adequate therapy for individual patients is essential. In this vein, it should be emphasised that the mechanisms underlying the development of each cancer vary, and a consideration of the mechanistic parameters mirroring the different molecular pathways is required for tailored cancer therapy.

We have confirmed that CIN is prevalent in ovarian cancer cell lines (Figure 1), consistent with the notion that taxane, the microtubule poison, has factored to a large extent in the treatment of ovarian cancers. As the mutations in SAC genes are rare (Cahill *et al*, 1998; Imai *et al*, 1999; Haruki *et al*, 2001; Hernando *et al*, 2001; Olesen *et al*, 2001; Langerod *et al*, 2003; Yuan *et al*, 2006), it seems that examining the levels of SAC proteins may be more informative. Furthermore, despite the incidence of CIN in ovarian cancers, the status of SAC proteins such as Bub1, Mad2, Mad1, and BubR1 had not been satisfactorily addressed (Lee *et al*, 2004; Fu *et al*, 2007). With these notions, we analysed the levels of BubR1 and Aurora A in 170 specimens for their clinical significance. The results showed a striking correlation between BubR1 levels and RFS. Moreover, a high level of BubR1 was associated with several aggressive clinicopathological parameters. These data imply the possibility that BubR1 may be related to the progression of epithelial ovarian cancers. Related to our study, an overexpression of BubR1 has been observed in several human cancers, including breast, gastric, bladder, kidney, and colorectal carcinomas (Myrie *et al*, 2000; Shichiri *et al*, 2002; Grabsch *et al*, 2003; Yamamoto *et al*, 2007; Pinto *et al*, 2008). In addition, it has been reported that the overall pattern of BubR1 localisation, revealed by immunostaining, differed between normal and malignant tissues in bladder, colon, pancreas, and skin cancers (Shin *et al*, 2003; Yamamoto *et al*, 2007; Burum-Auensen *et al*, 2007b).

Functional BubR1 is crucial for dividing cells, but not for quiescent cells. Therefore, our results showing that BubR1 elevation is correlated with poor prognosis, cancer aggression, and RFS may reflect the proliferative capacity of tumour cells. It is very likely that the expression of BubR1 is elevated in cycling cells (Burum-Auensen *et al*, 2007b). Therefore, we speculate that the elevated level of BubR1 coincides with a high mitotic index. We do not think that BubR1 elevation initiates tumourigenesis. Instead, we speculate that mutations in genes responsible for proliferative cell signaling or in tumour suppressor genes such as *BRCA1*, *BRCA2*, and *p53* are likely to take place before the elevation of BubR1 levels. Continued proliferation will make the cells susceptible to a high mutation rate, accompanied by massive cell death. Surviving cells are likely to have higher levels of BubR1 owing to forced proliferation, and at the same time acquire genetic instability because of accumulated mutations. This will accelerate the heterogeneity of the tumour, resulting in resistance to conventional cancer therapy.

Our finding that Aurora A is not a prognostic factor in ovarian cancers contradicts the report that amplification of Aurora A has been observed in many human cancers. Despite reports that the overexpression of Aurora A in Rat1 or NIH3T3 cells induces transformation (Bischoff *et al*, 1998; Zhou *et al*, 1998), transgenic mice overexpressing Aurora A (Zhang *et al*, 2004) or the overexpression of Aurora A in normal fibroblasts (Anand *et al*, 2003) does not induce neoplastic transformation. These seemingly contradictory results may come from the fact that the immortalised cell lines, such as Rat1 or NIH3T3, used by Bischoff *et al* (1998) and Zhou *et al* (1998), already harbour mutations and are, therefore, easier to transform. By comparison, Aurora A overexpression does not induce tumourigenesis in primary fibroblasts and mice. Thus, our results may correlate with the notion that amplification of Aurora A may result in CIN, whereas overexpression by itself does not initiate neoplastic transformation (Giet *et al*, 2005). Similarly, BubR1 amplification does not initiate tumourigenesis, and its amplification has not been reported in human cancers.

In conclusion, we suggest that BubR1 is a reliable prognostic marker for predicting RFS after initial treatment, and that assessing the levels of BubR1 in early-stage ovarian cancer patients, especially with FIGO I stage, can influence the choice of adequate treatment for ovarian cancers. Furthermore, modulating BubR1 activity may be a promising future approach to tailored therapy for ovarian cancers.

## Figures and Tables

**Table 1:** Clinicopathological characteristics of ovarian cancer patients

	**Number of patients (%)**
Median age (year, range)	51.5 (18–79)
*FIGO stage*	
I	43 (26.9)
II	13 (8.1)
III	86 (53.8)
IV	18 (11.2)
	
*Histology*	
Serous	92 (57.5)
Mucinous	24 (15.0)
Endometrioid	23 (14.4)
Clear cell	18 (11.2)
Others	3 (1.9)
	
*Grade*	
1	27 (16.9)
2	43 (26.9)
3	90 (56.2)
	
*Residual tumour*	
Residual tumour ⩽1 cm	83 (51.9)
Residual tumour >1 cm	77 (48.1)
	
*Clinical response*	
Complete response	111 (69.4)
Partial response	16 (10.0)
Stable disease	15 (9.4)
Progressive disease	18 (11.2)

**Table 2:** Score of BubR1 and Aurora A levels in ovarian tissue samples

	**Ovarian cancer (*n*=160)**		**Normal ovary (*n*=10)**	
	**BubR1 (%)**	**Aurora A (%)**	**BubR1 (%)**	**Aurora A (%)**
Score 0	39 (24.4)	45 (28.1)	10 (100.0)	10 (100.0)
Score 1	60 (37.5)	63 (39.4)	0	0
Score 2	42 (26.3)	36 (22.5)	0	0
Score 3	19 (11.9)	16 (10.0)	0	0

**Table 3:** Correlation of BubR1 level and clinicopathological parameters

		**BubR1 expression**		
	**Number of patients**	**Negative**	**Positive**	***P*-value**
*Age*				
<55 years	97	28	69	0.016
⩾55 years	63	11	52	
				
*FIGO stage*				
Early (I, II)	56	26	30	0.001
Advanced (III, IV)	104	13	91	
				
*Histology*				
Serous	92	13	79	0.008
Non-serous	68	26	42	
				
*Grade*				
1	27	16	11	0.001
2 and 3	133	23	110	
				
*Residual tumour*				
⩽1 cm	83	32	51	0.021
>1 cm	77	7	70	

**Table 4:** Univariate and multivariate analysis of prognostic factors in ovarian cancer patients for recurrence-free survival

	**Univariate analysis**	**Multivariate analysis**	
	***P*-value**	***P*-value**	**RR (95% CI)**
*Age*			
<55 years *vs* ⩾55 years	0.153	0.134	1.506 (0.085–2.261)
*FIGO stage*			
Early (I, II) *vs* advanced (III, IV)	<0.001	0.001	4.375 (2.920–6.555)
*Histology*			
Serous *vs* non-serous	<0.001	0.045	0.363 (0.240–0.549)
*Grade*			
1 *vs* 2 and 3	0.004	0.012	2.269 (1.686–3.053)
*Residual tumour*			
⩽1 cm *vs* >1 cm	<0.001	0.006	3.197 (1.905–5.364)
*BubR1 expression*			
Negative *vs* positive	<0.001	0.035	1.929 (1.400–2.656)

CI=confidence interval; RR=relative risk.
