# Supplementary figures and images for: BubR1 as a prognostic marker for recurrence-free survival rates in epithelial ovarian cancers
Source: Br J Cancer. 2009 Jul 14;101(3):504–10. doi: 10.1038/sj.bjc.6605161 (PMC2720250; doi:10.1038/sj.bjc.6605161)

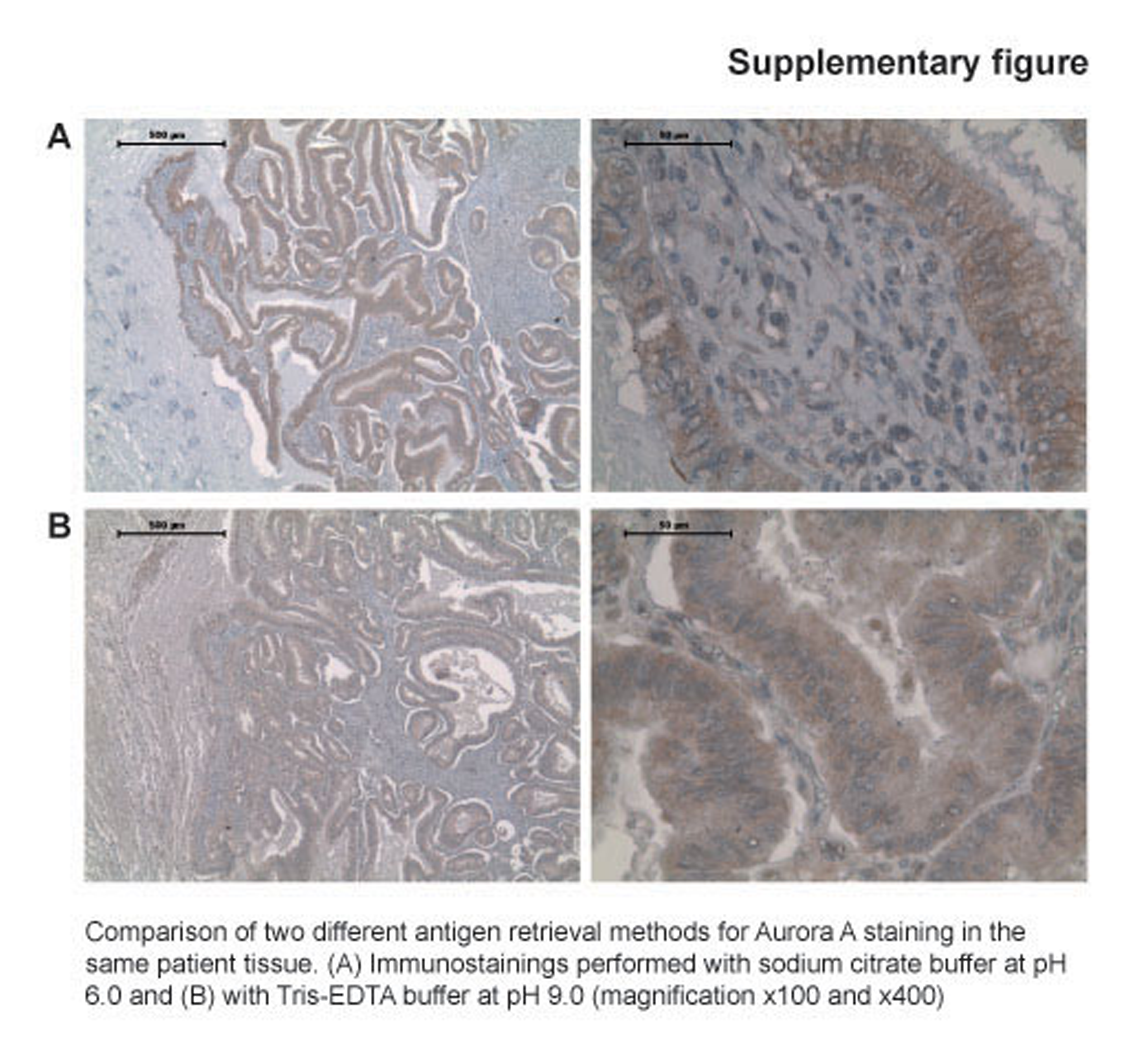

Supplement: Supplementary Figure [file 6605161x1.tif]
